# Descending Colon Epiploic Appendagitis Mimicking Diverticulitis: A Case Report

**DOI:** 10.7759/cureus.102484

**Published:** 2026-01-28

**Authors:** Anas E Ahmed, Omar G Alahmadi, Abdullah H Althurwi, Osaid H Niazi, Yazen A Alomary

**Affiliations:** 1 Community Medicine, Jazan University, Jazan, SAU; 2 College of Medicine, Jeddah University, Jeddah, SAU

**Keywords:** acute abdominal pain, case report, computed tomography, conservative management, descending colon, diverticulitis mimic, epiploic appendagitis

## Abstract

Epiploic appendagitis is a rare, self-limiting cause of acute abdominal pain that often mimics more common conditions, such as diverticulitis, leading to frequent misdiagnosis and unnecessary interventions. We report a case of a 46-year-old male who presented with sudden-onset, sharp, localized left lower quadrant pain without systemic symptoms. Physical examination revealed localized tenderness without peritoneal signs, and laboratory tests showed only mild leukocytosis and slight elevation of inflammatory markers. Contrast-enhanced computed tomography demonstrated an oval, fat-density lesion adjacent to the descending colon with a hyperattenuating rim and surrounding fat stranding, consistent with epiploic appendagitis. The patient was managed conservatively with analgesia and supportive care, with rapid symptom resolution within 48 h and complete recovery at two-week follow-up. This case highlights the importance of considering epiploic appendagitis in the differential diagnosis of acute abdominal pain, particularly when systemic signs are minimal, and demonstrates the value of imaging in establishing a definitive diagnosis. Prompt recognition can prevent unnecessary antibiotics, hospitalization, or surgical intervention, allowing for safe and effective conservative management.

## Introduction

Epiploic appendagitis is an uncommon, self-limiting cause of acute abdominal pain, resulting from inflammation of the epiploic appendages - small, fat-filled peritoneal pouches attached to the colon [1,2]. The condition most frequently affects the sigmoid and descending colon, although it can occur along any segment of the large intestine [2,3]. Clinical presentation often mimics more common acute abdominal pathologies, such as diverticulitis, appendicitis, or omental infarction, leading to frequent misdiagnosis and unnecessary interventions [3,4]. Patients typically present with sudden-onset, localized abdominal pain, minimal systemic symptoms, and normal or mildly elevated inflammatory markers, which further complicates clinical assessment based on history and examination alone [3,5].

Advances in imaging, particularly contrast-enhanced computed tomography (CT), have greatly improved the ability to accurately diagnose epiploic appendagitis, allowing for non-invasive management and avoidance of unnecessary hospitalization or surgery [3,6]. CT typically demonstrates a characteristic fat-density oval lesion adjacent to the colon with surrounding inflammatory stranding and a hyperattenuating rim, without colonic wall thickening. Awareness of this condition among clinicians is crucial to distinguish it from diverticulitis and other surgical emergencies, ensuring appropriate conservative management with analgesia and close follow-up. Reporting such cases enhances understanding of their clinical spectrum and imaging features, contributing to improved diagnostic accuracy and patient outcomes.

## Case presentation

A 46-year-old male presented to the emergency department with acute-onset left lower quadrant abdominal pain that began approximately 24 h before admission. The pain was described as sharp, constant, and localized, with no radiation. The patient reported associated mild nausea but denied vomiting, diarrhea, constipation, hematochezia, melena, fever, chills, or urinary symptoms. He had no history of similar abdominal pain, prior abdominal surgeries, or chronic gastrointestinal disorders. His past medical history was notable only for well-controlled hypertension. He denied alcohol use, smoking, or recent travel, and there was no family history of colorectal cancer or inflammatory bowel disease. The patient reported adherence to a regular diet and no recent changes in bowel habits.

On presentation, the patient appeared in mild discomfort but was alert and oriented. Vital signs revealed a blood pressure of 132/78 mmHg, heart rate of 88 beats per minute, respiratory rate of 18 breaths per minute, temperature of 37.1°C, and oxygen saturation of 98% on room air. Physical examination demonstrated localized tenderness over the left lower quadrant with mild voluntary guarding. There was no rebound tenderness, rigidity, or signs of peritonitis. Bowel sounds were normal, and examination of other abdominal quadrants was unremarkable. There was no costovertebral angle tenderness, and the remainder of systemic examination, including cardiovascular, respiratory, and neurologic systems, was within normal limits.

Laboratory investigations revealed a white blood cell count of 10.2×10^9^/L with mild neutrophilia (76%), hemoglobin of 14.2 g/dL, and platelet count of 230×10^9^/L. C-reactive protein was mildly elevated at 12 mg/L. Renal and liver function tests were within normal limits, and urinalysis was unremarkable. Given the localized pain and laboratory findings suggestive of a mild inflammatory response, a contrast-enhanced computed tomography (CT) scan of the abdomen and pelvis was performed to evaluate for diverticulitis or other acute abdominal pathology.

**Figure 1:**
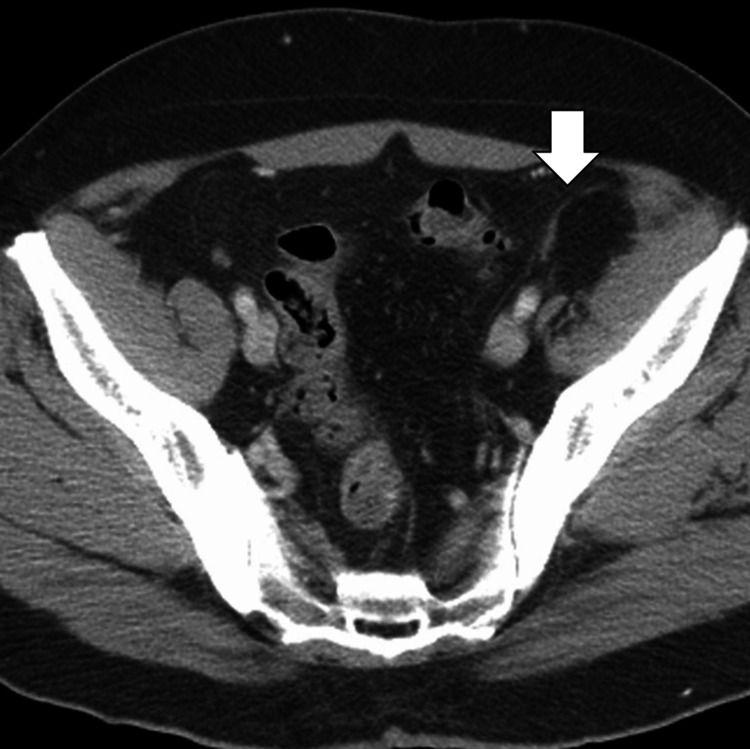
Axial contrast-enhanced CT image of the abdomen demonstrating epiploic appendagitis (arrow) adjacent to the descending colon. Note the characteristic fat-stranding and the "central dot" sign, representing a thrombosed vessel within the inflamed appendage.

**Figure 2:**
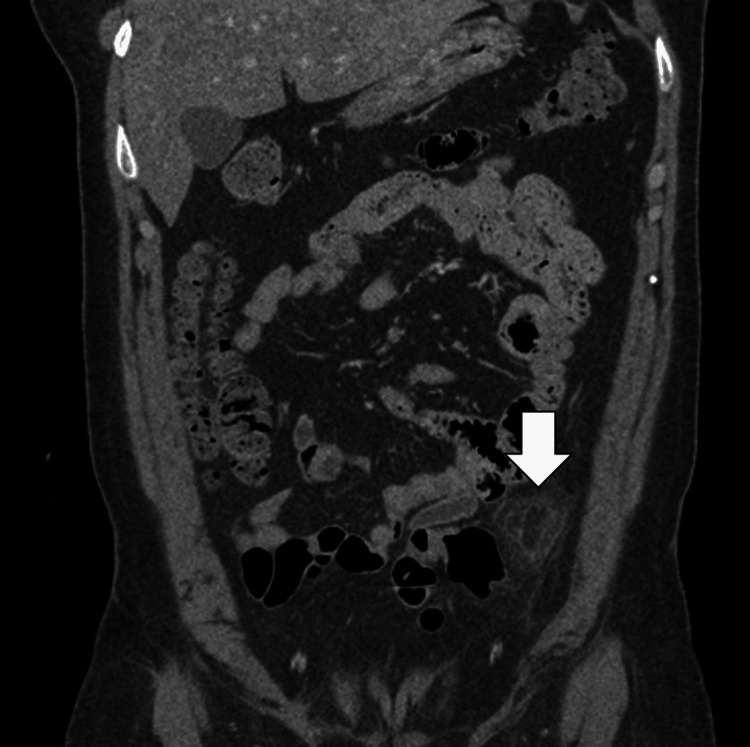
Coronal reformatted CT image showing an oval, fat-density lesion (arrow) along the serosal surface of the descending colon, consistent with acute epiploic appendagitis. There is no evidence of associated bowel wall thickening.

CT imaging demonstrated an oval, fat-density lesion measuring approximately 2.5 cm adjacent to the descending colon with a hyperattenuating rim and surrounding inflammatory fat stranding. There was no evidence of colonic wall thickening, abscess formation, free intra-peritoneal air, or other acute intra-abdominal pathology. These findings were consistent with epiploic appendagitis of the descending colon. No diverticula or signs of diverticular inflammation were observed (Figures 1, 2).

The differential diagnosis initially included acute diverticulitis, omental infarction, mesenteric panniculitis, and, less likely, intra-abdominal malignancy or ischemic colitis. The absence of colonic wall thickening, lack of systemic signs of infection, and characteristic imaging features supported a diagnosis of epiploic appendagitis. The patient was managed conservatively with non-steroidal anti-inflammatory drugs (NSAIDs) for analgesia and instructed to maintain oral hydration and a regular diet as tolerated. No antibiotics were administered, given the non-infectious nature of the condition.

During hospitalization, the patient’s symptoms gradually improved over 48 h. Vital signs remained stable, and serial abdominal examinations showed decreasing tenderness. Repeat laboratory tests after 24 h demonstrated stable leukocyte counts and normalization of C-reactive protein. The patient was discharged on day two with advice on symptom monitoring and instructions to seek medical attention if pain recurred or worsened. At two-week follow-up, he reported complete resolution of abdominal pain, resumed normal activities, and had no recurrence of symptoms.

## Discussion

Epiploic appendagitis is an infrequent and often underrecognized cause of acute abdominal pain, with an estimated incidence of 8.8 cases per million population per year [3,5]. It predominantly affects adults between 40 and 50 years of age, with a slight male predominance [4-6]. The condition results from either torsion of an epiploic appendage or spontaneous thrombosis of its central draining vein, leading to localized ischemia, inflammation, and necrosis [1,3]. Although epiploic appendages are distributed along the entire colon, the sigmoid colon is the most commonly involved segment, followed by the descending colon [3,7]. The clinical presentation is usually acute, localized, non-migratory pain without systemic signs of infection, which can closely mimic more prevalent conditions, such as acute diverticulitis or appendicitis, depending on the colonic segment involved [1,6]. This mimicry contributes to frequent misdiagnosis, unnecessary antibiotic use, and in some cases, unwarranted surgical intervention.

Accurate diagnosis relies primarily on imaging, as laboratory investigations are often non-specific, with only mild leukocytosis or inflammatory marker elevation in some cases [3,5]. Contrast-enhanced computed tomography (CT) is considered the diagnostic modality of choice, demonstrating a pathognomonic ovoid, fat-density lesion with surrounding inflammatory stranding and a hyperattenuating rim adjacent to the colon. Importantly, the absence of colonic wall thickening and extraluminal air helps differentiate epiploic appendagitis from diverticulitis, omental infarction, or ischemic colitis [2,4,6]. Ultrasound can occasionally identify a non-compressible, hyperechoic mass with a surrounding hypoechoic rim, though operator dependence limits its reliability [1,6]. Recognizing these radiologic features is crucial, as it allows for conservative, non-surgical management, which typically includes analgesics, anti-inflammatory medications, and supportive care, with symptoms resolving within one to two weeks in most patients [2,5]. Surgical intervention is rarely required and is reserved for persistent or complicated cases, underscoring the importance of early, accurate imaging-based diagnosis.

From a clinical standpoint, this case highlights the importance of including epiploic appendagitis in the differential diagnosis of left lower quadrant pain, particularly when laboratory findings and systemic signs are disproportionately mild relative to the severity of localized tenderness [7,8]. Misdiagnosis as diverticulitis can lead to unnecessary hospitalization, intra-venous antibiotics, and even surgical exploration, underscoring the need for heightened clinical awareness. Moreover, reporting cases of epiploic appendagitis involving less commonly affected segments, such as the descending colon, enriches the existing literature and aids in refining diagnostic algorithms for acute abdominal pain [3,6,8]. Future studies aimed at evaluating long-term outcomes, recurrence rates, and the cost-effectiveness of conservative management strategies could further inform clinical practice. Overall, this case reinforces the principle that careful correlation of clinical presentation with characteristic imaging findings is essential for optimal patient management and avoidance of unwarranted interventions.

## Conclusions

In summary, epiploic appendagitis, though rare, should be considered in the differential diagnosis of acute, localized abdominal pain, particularly when systemic signs and laboratory abnormalities are minimal. Accurate recognition, primarily through characteristic CT imaging features, is essential to distinguish it from more common surgical emergencies such as diverticulitis, thereby preventing unnecessary antibiotic therapy, hospitalization, or surgical intervention. Conservative management with analgesia and supportive care is typically sufficient, with rapid symptom resolution and excellent outcomes. This case underscores the importance of clinical vigilance and the integration of imaging findings into decision-making, emphasizing that awareness of epiploic appendagitis can optimize patient care and reduce healthcare burden.
